# Genomic epidemiology of syphilis in England: a population-based study

**DOI:** 10.1016/S2666-5247(23)00154-4

**Published:** 2023-10

**Authors:** Mathew A Beale, Louise Thorn, Michelle J Cole, Rachel Pitt, Hannah Charles, Michael Ewens, Patrick French, Malcolm Guiver, Emma E Page, Erasmus Smit, Jaime H Vera, Katy Sinka, Gwenda Hughes, Michael Marks, Helen Fifer, Nicholas R Thomson

**Affiliations:** a Parasites and Microbes Programme, Wellcome Sanger Institute, Hinxton, UK; b Blood Safety, Hepatitis, STI & HIV Division, UK Health Security Agency, London, UK; c HCAI, Fungal, AMR, AMU and Sepsis Division, UK Health Security Agency, London, UK; d Brotherton Wing Clinic, Brotherton Wing, Leeds General Infirmary, Leeds, UK; e The Mortimer Market Centre, Central and North West London NHS Trust, London, UK; f Laboratory Network, Manchester, UK Health Security Agency, Manchester Royal Infirmary, Manchester, UK; g Virology Department, Old Medical School, Leeds Teaching Hospitals Trust, Leeds, UK; h Clinical Microbiology Department, Queen Elizabeth Hospital, Birmingham, UK; i Institute of Environmental Science and Research, Wellington, New Zealand; j Department of Global Health and Infection, Brighton and Sussex Medical School, University of Sussex, Brighton, UK; k Department of Infectious Disease Epidemiology, London School of Hygiene & Tropical Medicine, London, UK; l Faculty of Infectious and Tropical Diseases, London School of Hygiene & Tropical Medicine, London, UK; m Hospital for Tropical Diseases, University College London Hospitals NHS Foundation Trust, London, UK; n Division of Infection and Immunity, University College London, London, UK

## Abstract

**Background:**

Syphilis is a sexually transmitted bacterial infection caused by *Treponema pallidum* subspecies *pallidum*. Since 2012, syphilis rates have risen dramatically in many high-income countries, including England. Although this increase in syphilis prevalence is known to be associated with high-risk sexual activity in gay, bisexual, and other men who have sex with men (GBMSM), cases are rising in heterosexual men and women. The transmission dynamics within and between sexual networks of GBMSM and heterosexual people are not well understood. We aimed to investigate if whole genome sequencing could be used to supplement or enhance epidemiological insights around syphilis transmission.

**Methods:**

We linked national patient demographic, geospatial, and behavioural metadata to whole *T pallidum* genome sequences previously generated from patient samples collected from across England between Jan 1, 2012, and Oct 31, 2018, and performed detailed phylogenomic analyses.

**Findings:**

Of 497 English samples submitted for sequencing, we recovered 240 genomes (198 from the UK Health Security Agency reference laboratory and 42 from other laboratories). Three duplicate samples (same patient and collection date) were included in the main phylogenies, but removed from further analyses of English populations, leaving 237 genomes. 220 (92·8%) of 237 samples were from men, nine (3·8%) were from women, and eight (3·4%) were of unknown gender. Samples were mostly from London (n=118 [49·8%]), followed by southeast England (n=29 [12·2%]), northeast England (n=24 [10·1%]), and southwest England (n=15 [6·3%]). 180 (76·0%) of 237 genomes came from GBMSM, compared with 25 (10·5%) from those identifying as men who have sex with women, 15 (6·3%) from men with unrecorded sexual orientation, nine (3·8%) from those identifying as women who have sex with men, and eight (3·4%) from people of unknown gender and sexual orientation. Phylogenomic analysis and clustering revealed two dominant *T pallidum* sublineages in England. Sublineage 1 was found throughout England and across all patient groups, whereas sublineage 14 occurred predominantly in GBMSM older than 34 years and was absent from samples sequenced from the north of England. These different spatiotemporal trends, linked to demography or behaviour in the dominant sublineages, suggest they represent different sexual networks. By focusing on different regions of England we were able to distinguish a local heterosexual transmission cluster from a background of transmission in GBMSM.

**Interpretation:**

These findings show that, despite extremely close genetic relationships between *T pallidum* genomes globally, genomics can still be used to identify putative transmission clusters for epidemiological follow-up. This could be of value for deconvoluting putative outbreaks and for informing public health interventions.

**Funding:**

Wellcome funding to the Sanger Institute, UK Research and Innovation, National Institute for Health and Care Research, European and Developing Countries Clinical Trials Partnership, and UK Health Security Agency.

## Introduction

Syphilis is a sexually transmitted infection (STI) caused by the bacterium *Treponema pallidum* subspecies *pallidum* (hereafter referred to as *T pallidum*). Syphilis rates have been rising in many high-income countries since the beginning of the 21st century.^1^, ^2^, ^3^, ^4^ In England, new diagnoses of early syphilis (primary, secondary, and early latent) rose from 3011 in 2012 (5·6 per 100 000 population) to 8011 in 2019 (14·2 per 100 000 population).^3^ This increase has primarily been associated with gay, bisexual, and other men who have sex with men (GBMSM) engaging in high-risk sexual behaviours.^2^, ^3^, ^5^ However, cases of syphilis in heterosexual men and women have also risen, raising concerns about infection during pregnancy and risks of vertical transmission leading to congenital syphilis.^6^, ^7^ Between 2016 and 2019, annual syphilis diagnoses increased by 53% (from 660 to 1012) in men who identify as heterosexual and have sex with women (MSW) and 108% (from 294 to 614) in women who identify as heterosexual and have sex with men (WSM). 24 cases of congenital syphilis were identified in England between 2015 and 2020, 15 of which were in children born to mothers who tested negative at first trimester antenatal screening,^8^ indicating they had acquired syphilis later in pregnancy. Some cases were identified in regions across England with increases in syphilis among women and GBMSM, suggesting that overlapping sexual networks might have facilitated wider dissemination.^9^

**Research in context**

**Evidence before this study**

Detailed phylogenomic analyses investigating the epidemiology and transmission dynamics of *Treponema pallidum* are challenging due to low bacterial loads in clinical specimens, and difficulty in culturing the bacteria. We searched PubMed from Jan 1, 1998, to Aug 9, 2022, using the search terms “Syphilis” or “*Treponema pallidum*” and “genomic” or “genome(s)” or “sequencing”. We found 23 studies describing whole genome sequencing of the *T pallidum* subspecies *pallidum*, of which two used whole genome phylogenies to investigate sexual network epidemiology, with one large study of sexual networks done primarily in Victoria, Australia, which characterised two major circulating sublineages in that setting and putative sexual transmission networks with distinct sexual behavioural characteristics and potential bridging between networks.

**Added value of this study**

In this study, we linked national surveillance data to *T pallidum* genomes, and characterised the transmission dynamics of syphilis using samples from across the whole of England. Integration of national-level sociodemographic, spatiotemporal, and genomic data allowed the delineation of putative sexual networks at both the national and region levels, and revealed patterns not previously detected using epidemiological or genomic data alone.

**Implications of all the available evidence**

Our findings are consistent with findings in Australia that indicate that genomics can identify putative sociodemographic transmission clusters. However, in that study, genomic clusters included samples separated by multiple single nucleotide polymorphisms, which could represent several years of evolution. Our study explored the value of linking identical genomes, and highlights that, despite technical constraints, whole genome sequencing can be used to enable outbreak exclusion and identify putative local transmission clusters for epidemiological follow-up.

Although epidemiological surveillance provides insights into the rise in syphilis rates, this is not always sufficient. For example, a group of spatiotemporally clustered cases could represent a single outbreak and chain of transmission, but could also be the result of separate or unrelated transmission networks. Molecular typing methods^10^, ^11^, ^12^, ^13^ provide one possible way to supplement epidemiological observations by identifying genetically related *T pallidum* strains. However, these methods might not accurately reflect recent evolutionary relationships between strains,^14^, ^15^, ^16^ but instead cluster groups of bacteria that shared a common ancestor decades ago, meaning it would be impossible to accurately delineate strain clusters relevant to epidemiologically useful timelines (usually months to years).

Whole genome sequencing (WGS) has shown there are two co-circulating *T pallidum* lineages globally (Nichols and SS14),^17^, ^18^, ^19^ which can be further divided into 17 sublineages plus singletons.^17^ These data showed that *T pallidum* genomes accumulate single nucleotide polymorphisms (SNPs) very slowly, with a median molecular clock rate equivalent to one substitution per genome every 6·9 years (95% highest posterior density 5·9–8·2 years),^17^ similar to other studies,^20^, ^21^ which means that isolate genomes from strains circulating in the UK can be identical (zero pairwise-SNPs) to those from Canada, Australia, and other countries, and identical genomes were collected an average of 2·5 years apart (range 0–15 years). This genetic homogeneity has been suggested to indicate a global dissemination of *T pallidum* within the past 30 years, driven by a small number of multi-country sublineages,^17^ and that genomic approaches might also be of limited value to investigate or resolve epidemiological links between patients with syphilis.

Few studies have combined WGS with patient demographic and sexual behaviour metadata to explore epidemiological trends of *T pallidum*, with studies from Japan^22^ and Australia^21^ finding discrete genetic clusters associated with GBMSM and heterosexual people. We explored the value of WGS for supplementing existing epidemiological data for understanding transmission at national and regional levels. We combined detailed patient demographic and epidemiological data with WGS of *T pallidum* samples from England to gain insights into the different spatiotemporal and genomic transmission patterns of syphilis affecting GBMSM and heterosexual people.

## Methods

### Study design and participants

A detailed description of sample collection and patient metadata linkage in this population-based study is provided in appendix 1 (pp 1–2). *T pallidum*-positive genomic DNA samples were retrieved from historical archives (2012–17) held at the UK Health Security Agency (UKHSA, previously Public Health England) STI Reference Laboratory (Colindale, London), and prospectively collected (2016–18) from five laboratories with high syphilis caseloads who do in-house molecular *T pallidum* diagnostic testing (and thus do not usually refer to the UKHSA reference laboratory; Birmingham, Brighton, Leeds, London [Mortimer Market Clinic at Central and North West London NHS Trust], and Manchester).

For samples from UKHSA, patient metadata were obtained by linkage to the national STI surveillance system (Genitourinary Medicine Clinic Activity Dataset [GUMCAD]). For samples prospectively collected from the five non-referring laboratories, patient metadata available from local laboratory information systems was linked to GUMCAD data and integrated into the larger dataset after deduplication (appendix 1 pp 1–2). For comparison between the sequencing dataset and national surveillance rates, we also retrieved summary statistics from GUMCAD data for all patients with syphilis aged 16 years and older in England from 2012 to 2018 (n=50 845).

### Ethics and data governance

Ethical approval for all clinical samples was granted by the National Health Service (UK) Health Research Authority and Health and Care Research Wales (UK; 19/HRA/0112) and the London School of Hygiene & Tropical Medicine Observational Research Ethics Committee (16014). Diagnostic samples were identified at UKHSA using internal laboratory information systems. UKHSA has permission to process confidential patient data under regulation 3 (control of patient information) of the UK Health Service Regulations 2002. Information governance advice and ethics approval for this study were granted by the UKHSA Research Ethics and Governance Group. Full details of approvals and pseudonymisation of samples and patient metadata are described in appendix 1 (pp 2–3).

### Procedures

WGS of all clinical *T pallidum* samples used in this study has been previously described,^18^ and was done directly on the genomic DNA extracts from residual diagnostic samples using the pooled sequence capture method^19^ on Illumina HiSeq 4000. Detailed spatiotemporal and sociodemographic metadata retrieved from the national STI surveillance system were linked to the multiple sequence alignments and time-scaled phylogenies generated and validated previously, to maintain consistency (appendix 1 pp 1–5).^17^

### Outcomes

The primary outcome was to identify local lineages of *T pallidum* samples and distinguish transmission clusters by location or sexual orientation. A secondary outcome was to investigate rates and distribution of macrolide resistance-conferring alleles among *T pallidum* sublineages.

### Statistical analysis

All statistical analyses were done in R, version 4.1.2 (appendix 1 p 5).

### Role of the funding source

The funders of the study had no role in study design, data collection, data analysis, data interpretation, writing of the report, or decision to submit.

## Results

Between Jan 1, 2012, and Oct 31, 2018, we identified and submitted 497 samples considered suitable for sequencing, from which we recovered 240 genomes (198 from the UKHSA reference laboratory and 42 from other laboratories; appendix 1 p 9). Three duplicate samples (same patient and collection date) were included in the main phylogenies, but removed from further analyses of English populations, leaving 237 genomes. 220 (92·8%) of 237 samples were from men, nine (3·8%) were from women and eight (3·4%) were of unknown gender. 217 samples were grouped into nine official Public Health Regions; for 18 samples the region was unknown; two samples were referred to English laboratories from elsewhere in the UK (table). Most samples were from London (n=118 [49·8%]), followed by southeast England (n=29 [12·2%]), northeast England (n=24 [10·1%]), and southwest England (n=15 [6·3%]). Analysis of collection dates showed that although we had sequences from most regions throughout the study period, the northwest and Yorkshire and Humber were represented only at the beginning (2012 and 2013) and end (2018) of the timeline.

**Table:** Baseline characteristics of WGS and all syphilis diagnoses from GUMCAD in England, 2012–18

	**Whole genome sequencing (n=237)**	**All syphilis diagnoses (n=50 845)**
**Year**		
2012	21 (8·9%)	4856 (9·6%)
2013	19 (8·0%)	5308 (10·4%)
2014	12 (5·1%)	6342 (12·5%)
2015	38 (16·0%)	7351 (14·5%)
2016	71 (30·0%)	8034 (15·8%)
2017	48 (20·3%)	9177 (18·0%)
2018	28 (11·8%)	9777 (19·2%)
**Self-identified gender**		
Male	220 (92·8%)	42 168 (82·9%)
Female	9 (3·8%)	6467 (12·7%)
Unknown	8 (3·4%)	2210 (4·3%)
**Gender orientation**		
MSW	25 (10·5%)	8978 (17·7%)
GBMSM	180 (75·9%)	33 190 (65·3%)
WSM	9 (3·8%)	6467 (12·7%)
Men with unrecorded sexual orientation	15 (6·3%)	..
Unknown	8 (3·4%)	2210 (4·4%)
**Age group**		
16–24 years	30 (12·7%)	6194 (12·2%)
25–34 years	70 (29·5%)	16 298 (32·1%)
35–44 years	59 (24·9%)	13 487 (26·5%)
≥45 years	76 (32·1%)	14 671 (28·9%)
Unknown	2 (0·8%)	195 (0·4%)
**Region of residence**		
East Midlands	5 (2·1%)	2398 (4·7%)
East of England	8 (3·4%)	2495 (4·9%)
London	118 (49·8%)	24 326 (47·8%)
Northeast	24 (10·1%)	1497 (2·9%)
Northwest	6 (2·5%)	5079 (9·5%)
Southeast	29 (12·2%)	4776 (9·4%)
Southwest	15 (6·3%)	2082 (4·1%)
West Midlands	7 (3·0%)	3763 (7·4%)
Yorkshire & Humber	5 (2·1%)	2760 (5·4%)
Unknown	18 (7·6%)	1475 (2·9%)
UK (not England)	2 (0·8%)	194 (0·4%)
**UK birth status**		
Non-UK born	66 (27·8%)	24 191 (47·6%)
UK born	138 (58·2%)	26 654 (52·4%)
Unknown	33 (13·9%)	..
HIV status (GBMSM only)*		
Negative	121 (51·1%)	21 888 (43·1%)
Positive	65 (27·4%)	11 302 (22·2%)
Data unavailable	51 (21·5%)	17 655 (34·7%)
**Syphilis stage (matched to diagnoses only, n=140**†**)**		
Primary	114 (81·4%)	14 178 (27·9%)
Secondary	15 (10·7%)	9918 (19·5%)
Early latent	10 (7·1%)	12 261 (24·1%)
Late latent	1 (0·7%)	13 032 (25·6%)
Cardio	0	635 (1·3%)
Neuro	0	821 (1·6%)

Gender categories are based on reported sexual orientation and gender identity, not on behaviours. WGS=whole genome sequencing. GUMCAD=Genitourinary Medicine Clinic Activity Dataset. MSW=men who have sex with women. GBMSM=gay, bisexual, and other men who have sex with men. WSM=women who have sex with men.

* All diagnoses: n=33 190; WGS: n=186.

† WGS dataset only.

180 (75·9%) of 237 genomes came from GBMSM, compared with 25 (10·5%) from MSW, 15 (6·3%) from men with unrecorded sexual orientation, nine (3·8%) from WSM, and eight (3·4%) from people of unknown gender and sexual orientation (table; appendix 1 p 10). Notably, the most highly represented region (London) had a higher proportion of GBMSM (110 [93%]) compared with the next most highly represented regions (21 [72·4%] from the southeast, 15 [62·5%] from the northeast, and 12 [80·0%] from the southwest). Due to a low number of heterosexual individuals (30 of 195) in the UKHSA dataset, HIV status was restricted to GBMSM to prevent deductive disclosure (the prospectively collected samples included no MSW or WSM living with HIV). 65 (27·4%) of 237 samples in the national genome collection were from people living with HIV and these were distributed across seven of nine regions. In London, which had the highest proportion of GBMSM, 48 (40·7%) patients were living with HIV.

To establish how representative our national genome collection was of syphilis cases in England, we compared the distributions of sociodemographic characteristics of cases in the national genome collection with those from GUMCAD (table). Overall, our national genome collection (n=237) represented 0·5% of all syphilis diagnoses among patients aged 16 years and older in England during the period 2012–18 (n=50 845). Compared with diagnoses reported in GUMCAD, the samples used in the WGS project were broadly representative by age group, region of residence (including London *vs* non-London), and HIV status (GBMSM only). However, a greater proportion of cases in the genome collection were GBMSM (180 [75·9%] of 237 from the WGS project *vs* 33 190 [65·3%] of 50 845 in the general population), with fewer women (nine [3·8%] *vs* 6467 [12·7%]) and MSW (25 [10·5%] *vs* 8978 [17·7%]). The genome collection also had a much higher proportion of primary syphilis cases compared with GUMCAD (114 [81·4%] in the genome collection compared with 14 178 [27·9%] from GUMCAD), largely reflecting the clinical presentation of primary syphilis with ulcers that permit swabbing.

We inferred the presence of macrolide resistance-conferring SNPs in the ribosomal 23S as previously described,^18^ and found that 209 (88·2%) of 237 English genome samples carried the A2058G allele, six (2·5%) of 237 had an uncertain or mixed variant call at position 2058, and five (2·1%) of 237 carried the A2059G allele, meaning that only 17 (7·2%) of 237 English *T pallidum* genomes carried a wild type ribosomal 23S gene and were therefore predicted to be sensitive to macrolide antimicrobials.

**Figure 1:**
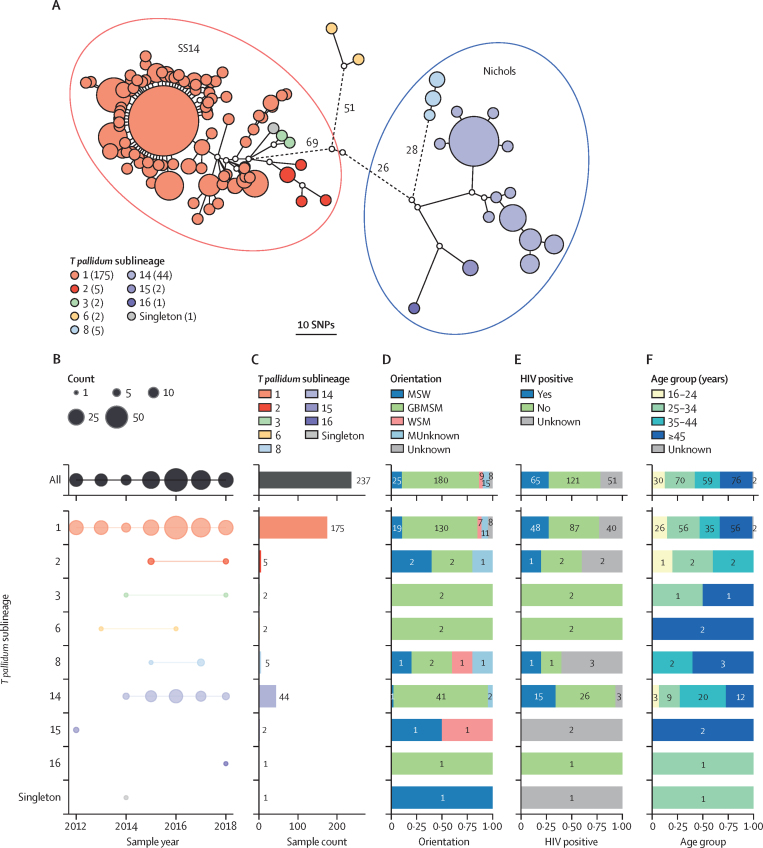
Population structure of English *Treponema pallidum* genomes according to phylogenetic sublineages and associated patient characteristics (A) Minimum spanning tree visualisation of genetic relationships between samples from England. Node size corresponds to the number of identical genome samples in a cluster, and edge length (with number) to the number of substitutions between identical genome sample clusters (where edges were longer than 12 substitutions, these have been shortened; this is indicated by dashed lines). Numbers in parentheses indicate total sample count for each sublineage. Primary lineage (SS14/Nichols) is indicated by encompassing ellipses; sublineage 6 diverges from other *Treponema pallidum* subspecies *pallidum* close to the root, and has previously been classified as Nichols.^18^ (B) Samples per collection year per sublineage. (C) Total sample counts per sublineage. Bar plots show proportion of each group. (D) Proportion of each group by sexual orientation. (E) Proportion of each group by HIV status. (F) Proportion of each group by age group (numbers indicate exact sample counts). SNP=single nucleotide polymorphism. MSW=men who have sex with women. GBMSM=gay, bisexual, and other men who have sex with men. WSM=women who have sex with men. MUnknown=men with unrecorded sexual orientation.

A whole genome phylogeny was inferred from the 237 English genomes sequenced here, along with 286 global contextual genomes. We clustered all isolates by lineage, sublineage, or into single-linkage SNP clusters.^23^ The English genomes were broadly distributed throughout the known *T pallidum* phylogeny (appendix 1 p 11). Referencing previous work by us and others,^17^, ^18^, ^19^ 183 (77·2%) of 237 genomes belonged to the SS14 lineage and 54 (23·8%) belonged to the Nichols lineage. Of the 17 defined global sublineages,^17^ eight were present in the UK, along with one singleton (figure 1A–C; appendix 1 p 12). The English samples were dominated by the global sublineages 1 (n=175 [73·8%]) and 14 (n=44 [18·6%]), but two other globally distributed sublineages (five from sublineage 2 and five from sublineage 8) were also detected in the UK (figure 1A, C) as well as two English samples for each of sublineages 3, 6, and 15, and one for sublineage 16.

Linking previously sequenced whole *T pallidum* genomes from English patients to STI surveillance and laboratory records, we observed that patients infected with the most common sublineage (175 [73·8%] of 237 from sublineage 1) were largely representative of syphilis patients overall, with 130 (74·3%) of 175 classified as GBMSM (figure 1D, appendix 1 p 12) and 91 (52·0%) aged 35 years or older. By contrast, 41 (93·2%) of 44 patients infected with sublineage 14 were GBMSM (1 of 44 MSW, 2 of 44 men with unrecorded sexual orientation), significantly more than would be expected by chance (Fisher's exact p=0·0087). 32 (72·7%) of 44 patients were aged 35 years or older, and 15 (34·1%) were living with HIV. We also found that most patients living with HIV were infected with sublineages 1 or 14, consistent with these lineages being linked to GBMSM networks (figure 1E, appendix 1 p 13). Seven (77·8%) of nine women were infected with *T pallidum* sublineage 1 and no women were infected with sublineage 14 (figure 1D, appendix 1 p 12). We found some rarer sublineage groups contained a greater proportion of heterosexual men and women, with sublineage 8 containing two heterosexual people (one [20·0%] of five WSM, one [20·0%] of five MSW), and sublineage 15 containing two heterosexual people (one [50·0%] of two WSM, one [50·0%] of two MSW) residing in the east of England. Analysis of the genetic relationships indicated that, at least in these two examples, the heterosexual samples were genetically identical to one another but distinct from other samples, falling on terminal nodes of our minimum spanning network (appendix 1 p 12).

To explore sociodemographic patterns of *T pallidum* genome clustering further, we delineated genomes from English patients into 27 distinct clusters of two or more genomes with zero pairwise-SNPs between them (ie, identical at the core genome level; appendix 1 p 14). Given the genetic homogeneity of *T pallidum*, these clusters do not necessarily indicate direct patient-to-patient transmission, but instead provided a means of clustering samples sharing a recent common ancestor. Of the 146 genomes included in these zero-distance genome clusters, 20 were from patients identifying as heterosexual (15 MSW and five WSM). 11 (55·0%) of these 20 patients were part of a cluster containing only other heterosexual individuals. Of the four genome clusters containing WSM, three contained only other heterosexual people and each of these clusters was detected in only a single region. However, the fourth cluster containing a WSM was the largest cluster of identical genomes, comprising 42 samples from sublineage 1, of which 35 patients (83·3%) were GBMSM, compared with three MSW, one WSM, and three with unknown sexual orientation. Therefore, this primarily GBMSM cluster also includes four patients who identify as heterosexual, indicating that there might be some bridging between the populations.

To understand the global context of *T pallidum* from England, we used a Bayesian time-scaled phylogeny of the global dataset (including contextual genomes from 21 other countries, appendix 1 p 15), and examined subtrees for the four most common English sublineages (1, 2, 8, and 14; appendix 1 p 16). Sublineage 1, previously found to be globally disseminated,^17^ contained samples from around England and the world; the English samples were polyphyletic and distributed throughout the sublineage 1 phylogeny, with little clustering (appendix 1 p 16), with the exception of a clade predominantly comprising samples from northeast England. Consistent with previous observations, 157 (89·7%) of 175 sublineage 1 strains carried the ribosomal 23S A2058G allele, and were predicted to be resistant to macrolides^17^, ^18^ (appendix 1 p 17). As previously described,^17^ sublineage 2 comprises two clades, one of which is dominated by North American samples, with the other dominated by samples from China. English samples were found within both clades, with at least three distinct groupings of English strains (appendix 1 p 16), probably indicating multiple recent independent introductions from other countries. By contrast, we found five sublineage 8 samples from England forming a monophyletic subclade along with individual samples from Canada and Australia (appendix 1 p 16). Sublineage 14 represented a major English sublineage, with 44 of 55 sublineage 14 genomes in the global collection coming from England, all of which had the ribosomal 23S A2058G allele. We previously described the contemporaneous appearance of this sublineage in England and Canada in 2013 to 2014,^17^ but our time-scaled phylogeny shows two clades within sublineage 14, both of which have median time to most recent common ancestors (1999 and 2006) predating the first detection in our dataset, suggesting this sublineage had been circulating in England for some time (appendix 1 p 16).

**Figure 2:**
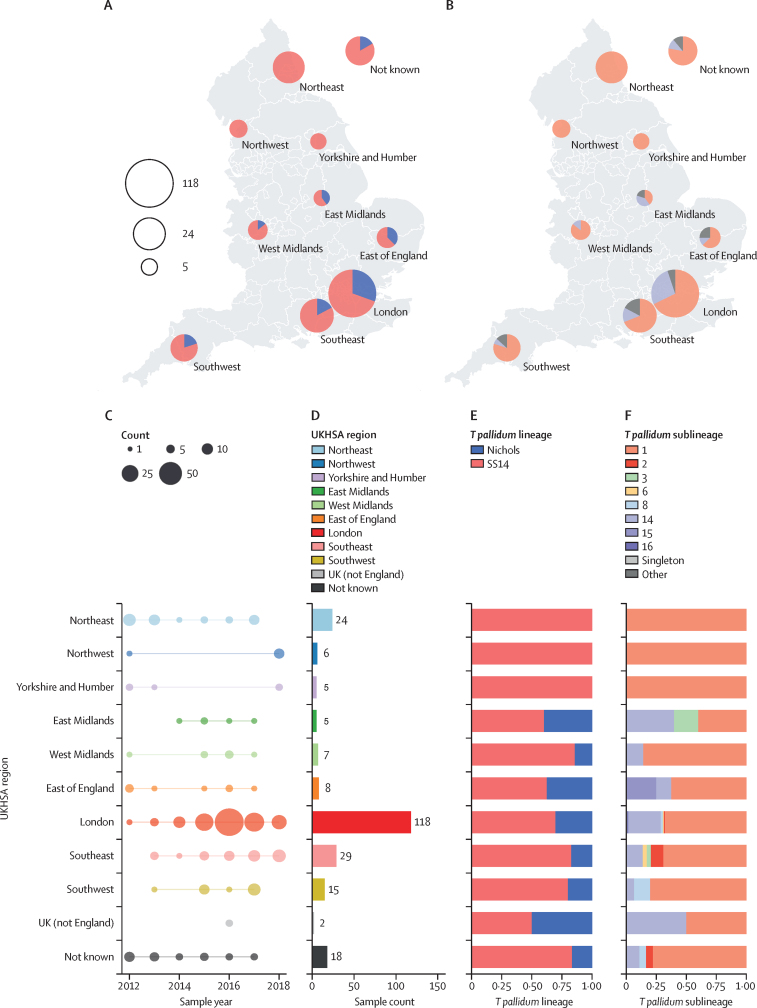
Geographical distribution of English genome samples and according to phylogenetic sublineages (A) Proportion of samples from each UKHSA region of England by *Treponema pallidum* subspecies *pallidum* lineage. (B) Proportion of samples in each public health region of England for the two most *common T pallidum* sublineages. (C) Distribution of sample collection years. (D) Total sample counts. (E) Proportion of samples from each region by lineage. (F) Proportion of samples from each region by sublineage. UKHSA=UK Health Security Agency.

We examined the geographical distribution of types in England and found that both the SS14 and Nichols lineages were co-circulating in London and throughout south and central England (figure 2A, E). However, we found only the SS14 lineage in the three most northerly regions (northeast, northwest, Yorkshire and Humber). Examination of *T pallidum* sublineage distributions indicated that sublineage 1 (SS14 lineage) was present in all regions (and represented the only sublineage present in the three northern regions), while sublineage 14 (Nichols lineage) was co-circulating with sublineage 1 in London, the south, and central England but not in northern England (figure 2B, F). There were 35 samples from the three northerly regions, of which 17 (48·6%) were collected after the first detected appearance of sublineage 14 in 2014, coinciding with an increase in national syphilis rates.^24^ From 2014 to 2018, the prevalence of sublineage 14 within the national genome collection was 22% (44 of 197). Under an assumption of even sample coverage and sublineage distributions, we could reasonably expect four samples (95% CI 1–7, p=0·022 for zero under a Poisson distribution) to be found in the northern regions, and the absence suggests regional strain distribution is not homogenous.

Apart from the three northern regions and the West Midlands (which contained only sublineages 1 and 14), all other regions contained at least three sublineages (range 3–7). 118 (49·7%) of 237 samples were from London, and we detected six sublineages (1, 2, 6, 8, 14, and 16) and one sublineage previously labelled as a singleton. As elsewhere, London was dominated by sublineages 1 (n=80, 67·8%) and sublineage 14 (n=32, 27·1%). The rare sublineages 15 (n=2) and 16 (n=1) were each found in a single region (sublineage 15 in the east of England and sublineage 16 in London), but all other sublineages were found in multiple regions (figure 2).

**Figure 3:**
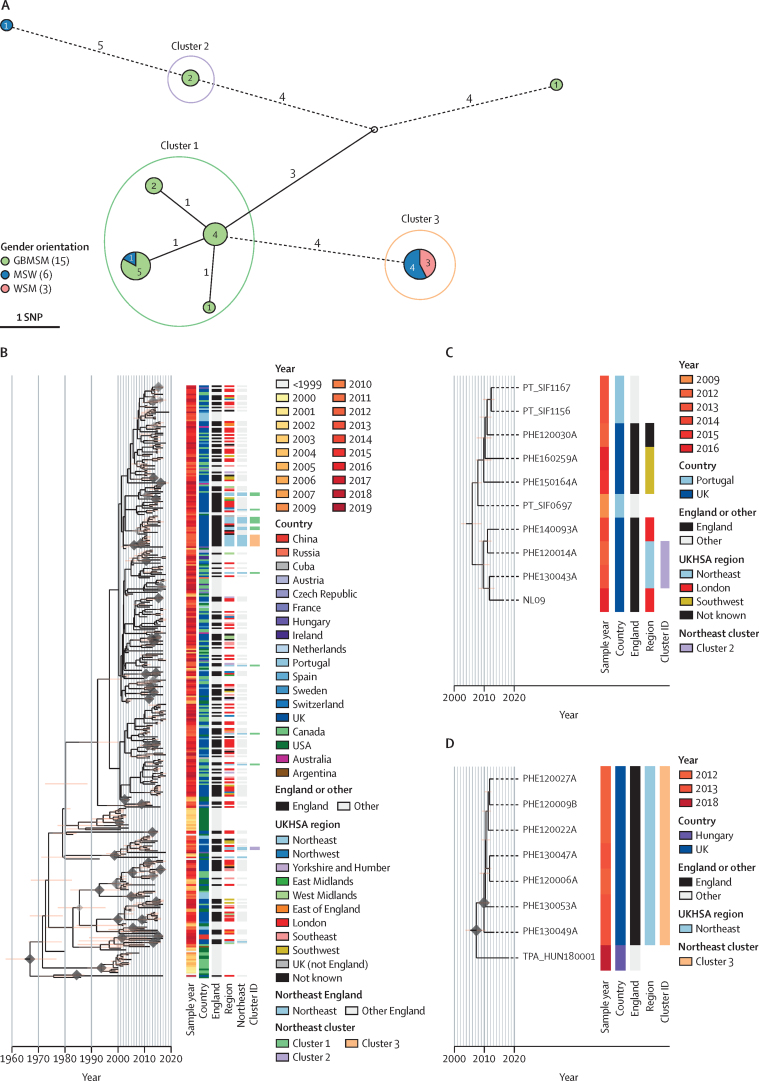
Spatiotemporal and genomic clustering analysis of samples from northeast England (A) Minimum spanning tree visualisation of genomic relationships between samples in northeast England. Node size corresponds to the number of identical samples, and edge length (with number) to the number of substitutions between clusters (where edges were longer than 3 substitutions, these have been shortened, indicated by dashed lines). Nodes are coloured by proportion of patient gender orientation). Clusters were defined by connections to another sample within two pairwise-single nucleotide polymorphisms. Clusters 1 and 2 appear to be dominated by GBMSM populations, while Cluster 3 contains only patients identifying as heterosexual. (B) Time-scaled sublineage 1 tree of global samples indicates that the GBMSM-associated cluster 1 is a globally distributed cluster, and that the northeast samples are polyphyletic. (C) Time-scaled subtree of global samples sharing a common ancestor with cluster 2 indicates a close relationship with two samples from London, and more distantly with those from southwest England and Portugal. (D) Time-scaled subtree of global samples sharing a common ancestor with the heterosexual-associated cluster 3 suggests the northeast England samples are closely related to each other, but not to any others, with the closest related strain found in Hungary. This could imply a historic importation from another country, followed by local circulation. MSW=men who have sex with women. GBMSM=gay, bisexual and other men who have sex with men. WSM=women who have sex with men. UKHSA=UK Health Security Agency.

Our geospatial analysis showed that all samples from the three most northerly regions of England were from SS14 sublineage 1 (figure 2), contrasting with greater diversity elsewhere in England. To explore this in more detail, we focused on the 24 samples collected from the northeast of England between 2012 and 2017. Although all samples belonged to sublineage 1, grouping northeast samples using a two pairwise-SNP threshold identified three distinct northeast clusters within sublineage 1 (figure 3A), and correlation with the global phylogeny (figure 3B; appendix 1 p 16) indicated different distributions (figure 3B). Cluster 1 comprised samples from 13 people that were collected between 2012 and 2017, 12 of which were samples from GBMSM. Within this cluster, samples could be further subdivided into three subgroups of eleven samples linked by identical core genomes (zero pairwise SNPs; figure 3A). Phylogenetic analysis of all English and global samples from sublineage 1 showed that, although posterior support for internal nodes was low, the northeast samples appeared to be polyphyletic (figure 3B) and interspersed with samples (including additional identical genomes) from the rest of England and around the world. Therefore, it is unlikely that the northeast samples from cluster 1 represent a direct chain of transmission or local outbreak, but rather that we have sampled from a broader transmission network spanning national and global boundaries.

Cluster 2 comprised two samples collected in 2012 and 2013 from GBMSM with identical core genomes and, similarly to cluster 1, formed a clade with eight other samples, of which five were from elsewhere in England (four of five GBMSM and one of five unknown) and three were from Portugal^25^ (figure 3C). By contrast, cluster 3 comprised seven samples, all of which came from the northeast in 2012 and 2013, and had identical core genomes (zero pairwise-SNPs). These samples formed a monophyletic clade, with the nearest related other sample in the global dataset separated by two SNPs and isolated from a bisexual man in Hungary in 2018 (figure 3D).^17^ All seven cluster 3 patients self-identified as heterosexual (MSW or WSM). Given the close spatiotemporal and genomic relationships between cluster 3 samples, and contextualised by a background of greater diversity over the 2012–17 timespan of all other samples collected from northeast England, cluster 3 probably represents a localised outbreak in a heterosexual network. This observation, made solely on the basis of the available genomic and sociodemographic data, is consistent with reports of a syphilis outbreak in heterosexual people in the northeast.^26^ It is likely that some of our samples are derived from this event.

## Discussion

In this study, we linked patient demographic, spatiotemporal, and behavioural metadata to previously generated *T pallidum* genomes from 237 patients diagnosed with syphilis in England between 2012 and 2018. Our analysis shows a variety of English sublineages, dominated by global sublineages 1 and 14,^17^ both of which are predicted to be resistant to macrolides, consistent with the high percentage of macrolide resistant samples in the UK. The English sublineages 1 and 14 displayed different patient sociodemographic and spatiotemporal profiles, with sublineage 1 patients showing a greater diversity of gender, sexual orientation, HIV status, and age, while sublineage 14 was primarily found in older GBMSM. Moreover, although sublineage 1 was found in all regions of England, cases attributed to sublineage 14 were mainly taken in London, and not found in the northern regions of England. These contrasting characteristics suggest that the two sublineages describe distinct sexual transmission networks, consistent with a recent WGS study from Australia,^21^ which identified broadly similar *T pallidum* population structures co-circulating in Melbourne and the Northern Territory. Both common sublineages (1 and 14) contained people living with and without HIV, and there was no phylogenetic delineation by HIV status, suggesting either that HIV status might not be strongly associated with transmission patterns, or that such patterns are beyond the ability of WGS-based analyses to detect.

We were also able to examine whether the data could be used to define GBMSM and heterosexual transmission networks based on the proportion of individuals identified as GBMSM or heterosexual for each genomic cluster.^21^ We observed three instances in which genomes from heterosexual individuals clustered with identical genomes only from other heterosexual people from the same region, consistent with this representing discrete heterosexual transmission networks or clusters. By contrast, we found that many genetic clusters classified as GBMSM-associated under a proportional definition across the whole dataset exhibited spatiotemporal diversity. These differences in spatiotemporal diversity could reflect differences in partner seeking behaviour and partner concurrency between GBMSM and heterosexual people.^27^ We also found mixed clusters, in particular a large cluster of 42 samples with identical core genomes, most of which were from GBMSM, four from heterosexual people (one woman, three men) and three with unreported sexual orientation or gender. Samples in this cluster had diverse regional geography and spanned across the 7 years of this study, and this implies widespread dissemination through the population more rapidly than the bacteria acquires variation, and potentially represents multiple local transmission networks all sharing a recent common ancestor. The presence of heterosexual people within these networks indicates possible bridging between transmission network groups.^28^

As in most countries, samples from England were dominated by sublineage 1.^17^, ^21^ Although most English sublineage 1 patients were GBMSM, with *T pallidum* genomes occupying positions across the sublineage 1 phylogeny and interspersed with samples from around the world, in the northeast of England we identified a genetically distinct cluster of identical core genomes found exclusively in heterosexual people, consistent with reports of a syphilis outbreak in heterosexual people at that time.^26^ Given the previous uncertainty as to whether genomics can have a substantial role in understanding the epidemiology of syphilis due to the genetic homogeneity and low molecular clock rate of *T pallidum*,^17^, ^20^, ^21^ our identification of discrete clusters associated with sexual behaviour suggests WGS combined with detailed epidemiological data can resolve some local transmission chains for *T pallidum*. This could offer opportunities to intervene or educate sexual networks, and to determine or exclude outbreak membership.

In other STIs, such as gonorrhoea, SNP cutoffs of either five or ten SNPs have been used to infer transmission chains.^28^, ^29^ *Neisseria gonorrhoeae* accumulates SNPs at a rate of eight substitutions per genome per year,^30^ nearly 60 times faster than *T pallidum*. Therefore, even *T pallidum* isolates with identical genomes do not necessarily indicate recent direct patient-to-patient transmission. Conversely, samples separated by even a very small number of SNPs are unlikely to share a recent common ancestor. Furthermore, because the potential transmission window of *T pallidum* might be as high as 2 years,^31^, ^32^ direct transmission cannot be excluded temporally for identical genomes collected within that period. This could ultimately limit our overall ability to deconvolute national or regional patterns of transmission.

Our study has several limitations, including the small number of samples compared to the total number of syphilis cases during the time period, and overrepresentation of samples from GBMSM in the national genome collection. The genomes represented 0·5% of the recorded number of syphilis cases in England during the study period and all samples referred to the National Reference Laboratory with sufficient treponemal DNA for sequencing. Although the available referral population might not be fully representative of syphilis in England due to regional variation in molecular testing and referral practices, all samples were collected and sequenced in the absence of any genetic relatedness information, so our genomic observations provide a snapshot of circulating English lineages. Future studies that focus on the systematic collection of samples from a higher proportion of cases, combined with improved sequence quality, will enable further insights into *T pallidum* transmission dynamics, and enable the fuller usefulness of sequence data to inform public health interventions.

## Data sharing

Sequencing reads for all genomes used in this study have been previously published and described, and are available at the European Nucleotide Archive (https://www.ebi.ac.uk/ena) in BioProjects PRJEB28546, PRJEB33181, and PRJNA701499. All accessions, corresponding sample identifiers, and related metadata are available in appendix 2. Patient metadata for the UK genomes is available in pseudonymised form in appendix 3. UK shape files for Public Health region boundaries were downloaded from the UK Office for National Statistics, available at https://geoportal.statistics.gov.uk. The R code for all phylogenetic and statistical analysis and plotting is available in an Rnotebook along with all sample metadata (pseudonymised) and intermediate analysis files at https://doi.org/10.6084/m9.figshare.21543333.v1 and https://github.com/matbeale/Syphilis_Genomic_Epi_England_2022-23.

## Declaration of interests

We declare no competing interests.
